# Supplementary material for: Real-time ultrafast oscilloscope with a relativistic electron bunch train
Source: Nat Commun. 2021 Nov 25;12:6851. doi: 10.1038/s41467-021-27256-x (PMC8617034; doi:10.1038/s41467-021-27256-x)
Supplement: Supplementary file 1 — Supplementary information. [file 41467_2021_27256_MOESM1_ESM.pdf]

Supplementary information

Real-time ultrafast oscilloscope with a relativistic electron bunch train

In Hyung Baek[1,*], Hyun Woo Kim[1,*], Hyeon Sang Bark[1,*], Kyu-Ha Jang[1], Sunjeong Park[1], Junho Shin[1], Young Chan Kim[1], Mihye Kim[1], Key Young Oang[1], Kitae Lee[1,2], Fabian Rotermund[3], Nikolay A. Vinokurov[4,5], and Young Uk Jeong[1,2]

[1]Korea Atomic Energy Research Institute (KAERI), Daejeon 34057, Republic of Korea

[2]University of Science and Technology (UST), Daejeon 34113, Republic of Korea

[3]Korea Advanced Institute of Science and Technology (KAIST), Daejeon 34141, Republic of Korea

[4]Budker Institute of Nuclear Physics SB RAS, Novosibirsk 630090, Russia

[5]Novosibirsk State University, Novosibirsk 630090, Russia

Correspondence to: Tel: +82-42-868-8342; Fax: +82-42-868-2969; e-mail: yujung@kaeri.re.kr

*These authors contributed equally to this work.

This file includes:

Figures 1 to 5

Equations (1) to (21)

Supplementary Note 1: Calculation of the effective length of field

Electromagnetic wave in the gap of the slit is the TEM one, see Supplementary Fig. 1.

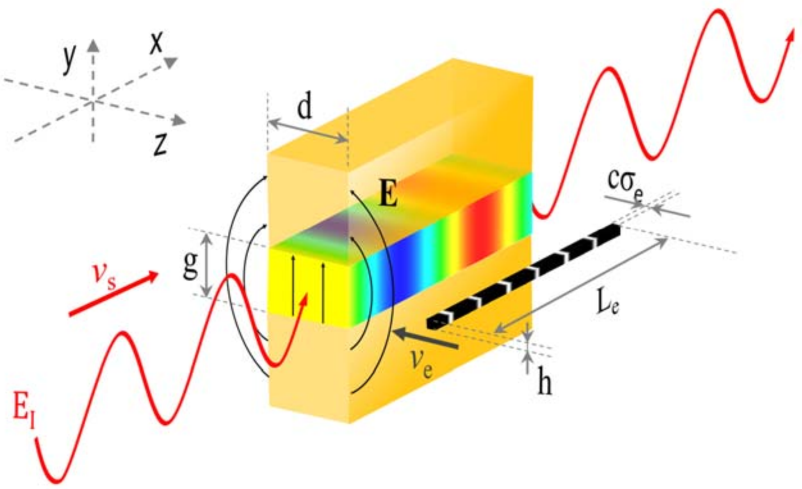

Supplementary Figure 1. The scheme of experiment. The 1D electron array meets the electric field (TEM mode) confined at gap.

It is easy to understand, as there is a two-wire line of two strips, and the gap g between strips is much less than the wavelength λ divided by 2π. The propagation direction of wave is x, therefore $E_{\mathrm{x}} = H_{\mathrm{x}} = 0$. Transverse components of electric field can be found from solution of 2-D electrostatic problem. For simple analytic estimate we will consider thick slit with gap g,$2\pi d/g$ >> 1 (see Supplementary Fig.2).

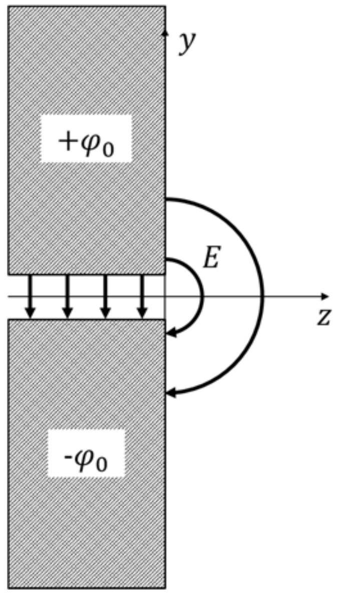

Supplementary Figure 2. Electric field at the edge of thick slit. The 1D electron array meets the electric field (TEM mode) confined at gap.

Then the corresponding potential $\varphi = \varphi_0 Im\Psi/\pi$ can be easily found using conformal mapping $z + iy = g\left(s - ln\sqrt{s+1} + ln\sqrt{s-1}\right)/\pi$, $s = \sqrt{e^{\Psi} + 1}$, where φ_0 is the potential of the top electrode. Then

$$E_y + iE_z = -\frac{\varphi_0}{\pi}\frac{d\psi}{d(z+iy)} = -\frac{2\varphi_0}{g}\frac{1}{s} = \frac{E_{max}}{s}, \qquad (1)$$

For |s| > 1 $s \approx \sqrt{\frac{\pi^2(z+iy)^2}{g^2} + 2}$ and

$$E_y + iE_z \approx \frac{g}{\pi}\frac{E_{max}}{\sqrt{(z+iy)^2 + 2g^2/\pi^2}}, \qquad (2)$$

Due to finite conductivity of the slit metal, which is described by Leontovich boundary conditions with the surface impedance ζ, the phase velocity of this wave is slightly lower than the speed of light in vacuum. It can be estimated using common formula for the attenuation in the waveguide

$$Imk_x = \frac{Re\zeta}{2}\int |H|^2 dx \,/ \int |H|^2 dydz \approx \frac{Re\zeta}{g}, \qquad (3)$$

In the first approximation k_x has to be linear function of the surface impedance, therefore

$$k_x \approx k + \zeta\frac{iIMk_x}{Re\zeta} \approx k + i\frac{\zeta}{g}. \qquad (4)$$

For metals with conductivity σ it gives

$$k_x \approx k + \frac{1+i}{g}\sqrt{\frac{ck}{8\pi\sigma}}. \qquad (5)$$

Then one of the limitations for the bandwidth of such oscilloscope can be expressed as

$$|k_x - k| x_{\max} \approx |\zeta| x_{\max}/g < 1, \qquad (6)$$

where $x_{\max}$ is the distance from the slit entrance. For 10 THz and copper slit $|\zeta|$ is about 10^{-3}. Therefore, $x_{\max} < 300g = 10\ mm$. It means that at shorter distances than 10 mm (from the waveguide entrance) there is no distortion for signals with a bandwidth of 10 THz in our waveguide.

Let electron coordinates are $[x, 0, v(t-t_1)]$. Then the transverse momentum variation of electron passed through the slit is

$$\Delta p_y = e\int_{-\infty}^{\infty} E_y(x, 0, vt - vt_1, t)dt$$

$$\approx \frac{e}{v}2E_{\max} cos\left[\omega(t_1 - \frac{x}{c})\right]\int_{-d/2}^{\infty}\frac{cos\left[\omega(z+\frac{d}{2})/v\right]}{s}dz$$

$$\approx \frac{e}{v}2E_{\max} cos\left[\omega(t_1 - \frac{x}{c})\right]\left[\left(\frac{2v}{\omega} - g\right)sin\left(\frac{\omega d}{2v}\right) + \frac{2g}{\pi}cos\left(\frac{\omega d}{v}\right)K_0\left(\frac{\omega g}{\pi v}\sqrt{2}\right)\right]$$

$$\approx \frac{e}{v}2E_{\max} cos\left[\omega(t_1 - \frac{x}{c})\right]\left[d + \frac{2g}{\pi}\left(ln\frac{\pi v\sqrt{2}}{\omega g} - C\right) - \frac{\omega g d}{2v}\right]$$

$$= \frac{eE_{\max}L_{\text{eff}}}{c}cos\left[\omega(t_1 - \frac{x}{c})\right], \tag{7}$$

where, K_0 is the modified Bessel function and L_{eff} is the effective length of the electric field in z-axis at the gap center of the slit waveguide.

$$L_{\text{eff}} \approx d + \frac{2g}{\pi}\left(ln\frac{\lambda}{g\sqrt{2}} - C\right) - \frac{\lambda g d}{\lambda}, \tag{8}$$

and $C \approx 0.577$ is Euler's constant. For $d = 25$ μm, $g = 30$ μm and $\lambda = 0.6$ mm, Eq. (8) gives $L_{\text{eff}} = 60$ μm. For $\lambda = 0.15$ mm, $L_{\text{eff}} = 35$ μm. Therefore, one can say that the bandwidth of the "oscilloscope" is about 1 THz.

The corresponding deflection y on the screen of the EMCCD is given by expression

$$y = \frac{\Delta p_y}{p}D = \frac{eDL_{\text{eff}}}{pc}E_0(t_1 - x/c), \tag{9}$$

where p is electron momentum, and D is the distance from the slit to the screen. Due to finite bunch duration σ_e, the line width in x coordinate is $c\sigma_e$. For $\sigma_e = 25$ fs, it is 7.5 μm, which corresponds to 2 pixels of the CCD.

The effective length of field, L_{eff} can be also found by FDTD simulation. Supplementary Fig. 3a shows the cross-sectional map (in *yz* plane) of in-gap electric field at distance of 2 mm from slit entrance. The incident wave has a pulse duration of 2 ps (FWHM) and a central frequency of 0.37 THz. This wave is coupled into the gap of Cu slit with g = 30 μm and d = 25 μm. Supplementary Fig. 2b shows the *z*-axis distribution of *y*-field depending on the *y*-position. From these results, we obtained the L_{eff} of 66.48 μm by using the formula of $\left(\int_{-\infty}^{\infty} E(y,z)dz\right)/E_{y,\max}$ at the median plane (y = 0), as shown in Supplementary Fig. 3c.

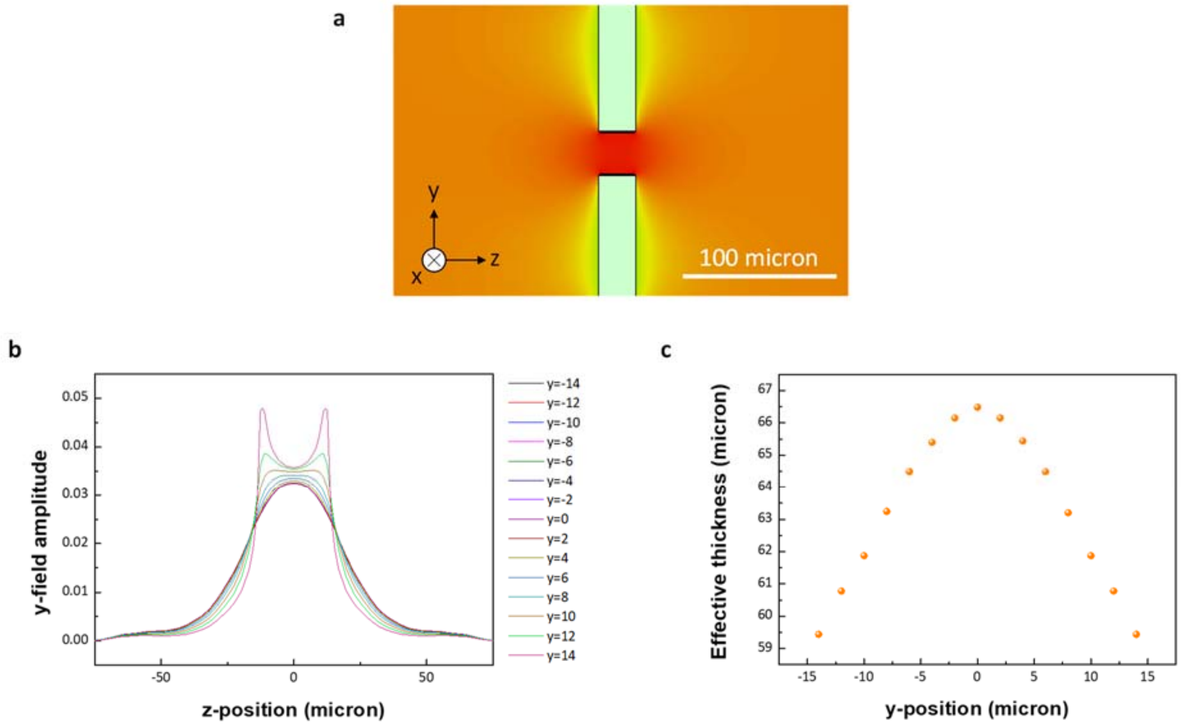

Supplementary Figure 3. FDTD simulation of the effective length of field. a, The vertical in-gap electric field distribution at distance of 2 mm from slit entrance. **b,** Plots for y-field distribution depending on y-position from -14 to 14. **c**, The calculation of L_{eff} as a function of y-position.

Supplementary Note 2: Calibration of the field measurement

Between the luminescent screen and CCD we have the objective lens, which provides image of the screen on the CCD with some magnification *M*. We can find *M* from measured correspondence of one pixel (32 microns) to 13.2 fs.

The TEM wave propagates along the copper slit at velocity *c*. Therefore, the pulse shift at 13.2 fs is 4 microns. It means that total magnification is

$$M\frac{D+l}{l}=\frac{32}{4}=8, \tag{10}$$

where D = 2.4 m is the distance from the slit to the screen, l is the distance from the beam horizontal waist to the slit. l can be found from the equation

$$\frac{1}{F}=\frac{1}{l_1}+\frac{1}{d-l}, \tag{11}$$

where F is the focal length of the magnetic lens, d = 0.57 m is the distance from the middle of the lens to the copper slit, and l_1 is the distance from the middle of the lens to the horizontal waist before the lens. Here we suppose the horizontal emittance to be small enough. As F = $1/kL$, where k is the focusing strength and L = 0.0834 m is the magnetic length of the lens, Eq. (11) can be rewritten as

$$kL=\frac{1}{l_1}+\frac{1}{d-l}. \tag{12}$$

Then l = 0 takes place at

$$k_0=\frac{1}{L}\left(\frac{1}{l_1}+\frac{1}{d}\right), \tag{13}$$

and one can rewrite Eq. (12) as

$$(k-k_0)L=\frac{1}{d-l}-\frac{1}{d}. \tag{14}$$

According to Fig. 4b in main text, $k - k_0$ = 20 m^{-2}. Then one can find from Eq. (10)

$$l=d-\frac{1}{(k-k_0)L+1/d}=\frac{d}{1+\left[(k-k_0)Ld\right]^{-1}}\approx .278\,\text{m}. \tag{15}$$

Then Eq. (10) gives

$$M=\frac{8}{1+D/l}=\frac{8}{1+\left(1+\frac{1}{(k-k_0)Ld}\right)\frac{D}{d}}\approx 0.83. \tag{16}$$

According to Fig. 2b in main text, maximum deflection corresponds to 61 pixels. If the vertical size of pixel is also 32 microns, maximum deflection angle is

$$\theta_{\max} = \frac{32\mu \cdot 61}{MD} \approx 0.98 \cdot 10^{-3} . \tag{17}$$

For pc = 3.6 MeV it gives

$$\int_{-\infty}^{\infty} E_y dz = \frac{pc}{e}\theta_{\max} \approx 3.5 \text{ kV} . \tag{18}$$

For effective length of field L_{eff} = 60 micron, $E_{\max} \approx$ 600 kV. It corresponds to the enhancement factor about 3.

Supplementary Note 3: Calculation of maximum THz field

Electric field of plane wave is

$$\left|E(t)\right| = \sqrt{\frac{I(t)}{c\varepsilon_0}} = \sqrt{\frac{P(t)}{c\varepsilon_0 2\pi\sigma_{THz}^2}} = \sqrt{\frac{P(t)}{c\varepsilon_0 2\pi\sigma_{THz}^2}},$$

where $I(t)$ is intensity, and $P(t)$ is power of Gaussian beam.

$$\sigma_{THz} = \frac{FWHM}{2\sqrt{2\ln 2}}$$

$$E_{\max} = \frac{2}{FWHM}\sqrt{\frac{P_{\max}\ln 2}{c\varepsilon_0\pi}} \tag{19}$$

is the maximum field.

$$P_{\max} = W\frac{E_{\max}^2}{\int_{-\infty}^{\infty} E^2 dt} = \frac{W}{T_{eff}}, \tag{20}$$

where $W = \int_{-\infty}^{\infty} P dt$ is the energy per pulse, $T_{eff} = \int_{-\infty}^{\infty} E^2 dt \Big/ E_{\max}^2$.

From (19) and (20) one has

$$E_{\max} = \frac{2}{FWHM}\sqrt{\frac{W\ln 2}{c\varepsilon_0\pi T_{eff}}}. \tag{21}$$

T_{eff} has to be calculated from measured EOS curve from Fig. 3a in main text.

For simple analytic estimate of T_{eff} one can take

$$E(t) = E_{\max}\sqrt{e}\frac{t}{\sigma}\exp\left(-\frac{t^2}{2\sigma^2}\right).$$

Then

$$T_{eff} = e\int_{-\infty}^{\infty}\left(\frac{t}{\sigma}\right)^2\exp\left(-\frac{t^2}{\sigma^2}\right)dt = \sigma\frac{e\sqrt{\pi}}{2} = \frac{T_{\max}-T_{\min}}{2}\frac{e\sqrt{\pi}}{2} =$$

$$\left(T_{\max}-T_{\min}\right)\frac{e\sqrt{\pi}}{4} \approx 1.2\left(T_{\max}-T_{\min}\right) \approx 1.2 \text{ ps},$$

where $T_{\max} - T_{\min} = 2\sigma \approx 1$ ps is the time interval between negative and positive peaks of the signal.

For W = 1.67 μJ and FWHM = 1 mm, Eq. (17) gives $E_{\max} = 215\ kV/cm$.

Supplementary Note 4: Electron beam emittance effect to waveform distortion

We carried out the simulation about electron beam emittance-induced blurring effects. All parameters except an electron beam divergence are same with the simulation in Methods section which describes the concept of this work. The initial horizontal divergence of electron beam is estimated as $x'_{ini} = \varepsilon_{N\_x}/\beta\gamma x_{ini}$, where ε_{N_x} is the normalized horizontal beam emittance, β is the relativistic velocity, γ is the Lorentz factor and x_{ini} is the horizontal electron beam size (rms). The initial vertical divergence also can be defined as $y'_{ini} = \varepsilon_{N\_y}/\beta\gamma y_{ini}$. Supplementary Fig. 4 shows the comparison of simulated THz waveforms whose beam emittances are applied or not. The THz waveform with a central frequency of 0.37 THz, a pulse duration of 2 ps, and an electric field strength of 25 MV/m was used as an input signal. The simulated waveform in Supplementary Fig. 4c is well matched with our experimental data (Fig. 2b) in main text.

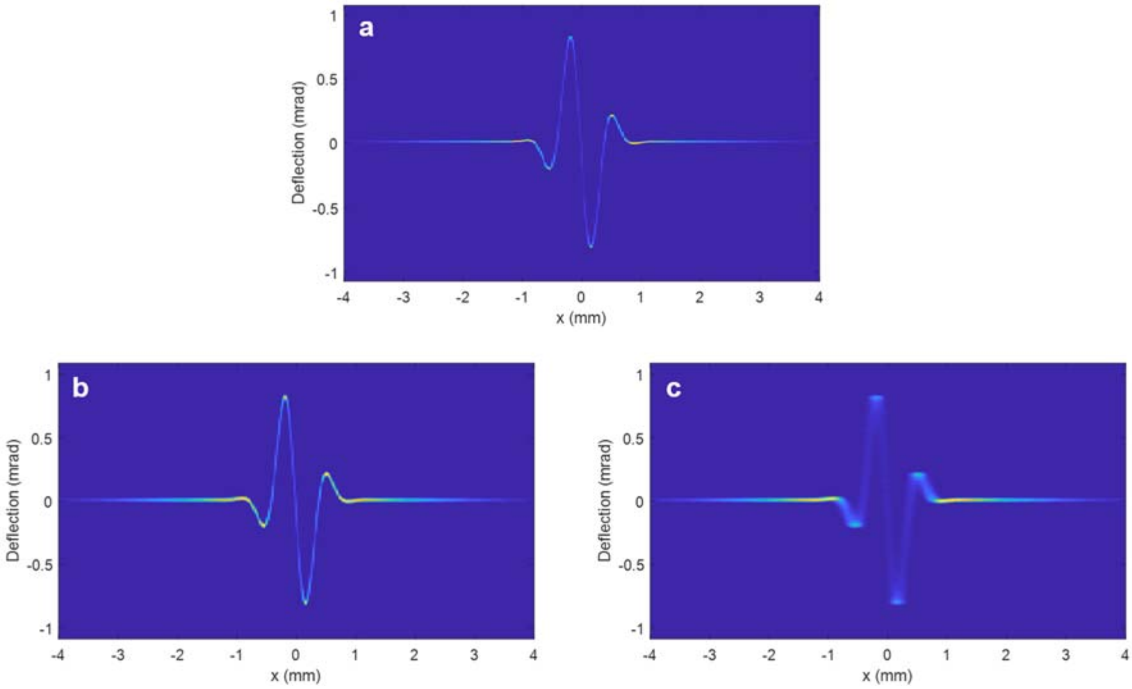

Supplementary Figure 4. THz waveform simulations depending on the electron beam emittance. a. Ideal case for $\varepsilon_{N\_x,y} = 0$, **b.** Only vertical emittance ($\varepsilon_{N\_y} = 0.3\ \mu m$) is considered. **c.** The identical emittance ($\varepsilon_{N\_x,y} = 0.3\ \mu m$) for both horizontal and vertical axes is applied for simulations.

To propose the applicability of our oscilloscope for extending its operation range, we performed a further simulation at NIR region. The peak electric field strength inside the slit is 150 MV/m, the central wavelength (λ) of input pulse is 800 nm, and the pulse duration is 5 fs (rms). Here, we changed the bunch length of 10^5 electrons and the normalized horizontal electron beam emittance at the fixed vertical one of 5.5 nm because the emittance effect at time-axis is to be seen clearly. For the electron bunch length of $\lambda/15$, the profile of waveform can be discerned even though ε_{N_x} is close to 90 pm which is corresponding to the horizontal divergence of 0.32 μrad. However, for the electron bunch length of $\lambda/4$, ε_{N_x} of 30 pm at least should be required to visualize a contour of waveform as shown in Supplementary Fig. 5b.

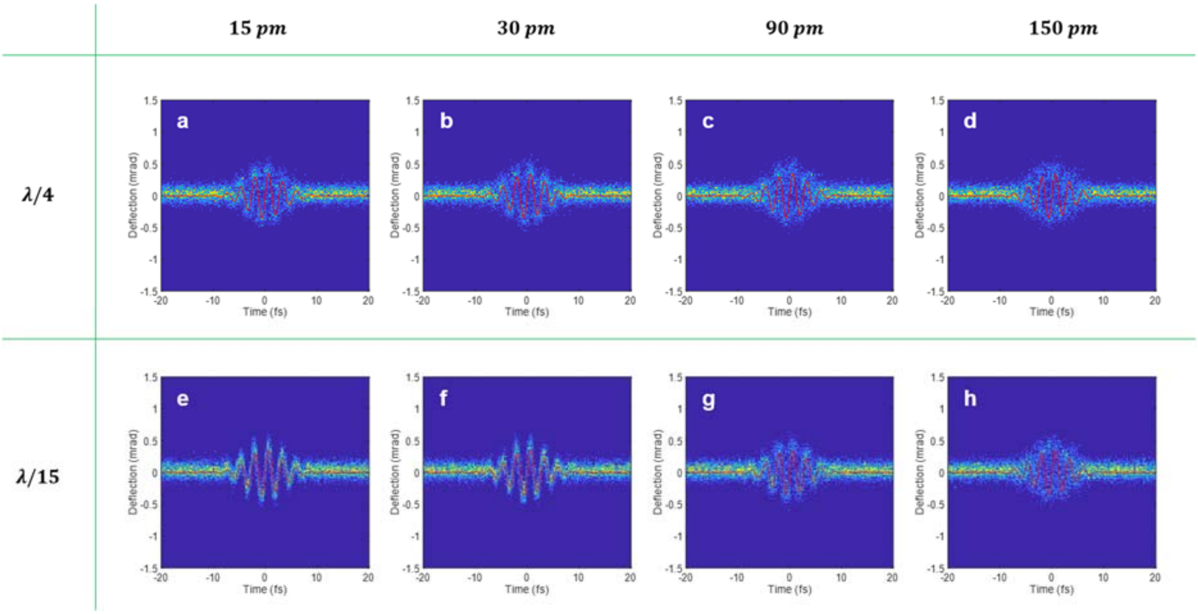

Supplementary Figure 5. NIR waveform simulations depending on the bunch length and emittance of electron beam.
